# Laboratory and field evaluation of the impact of washings on the effectiveness of LifeNet®, Olyset® and PermaNet® 2.0 in two areas, where there is a high level of resistance of *Anopheles gambiae* to pyrethroids, Benin, West Africa

**DOI:** 10.1186/1475-2875-13-193

**Published:** 2014-05-27

**Authors:** Fiacre R Agossa, Gil G Padonou, Virgile Gnanguenon, Frédéric Oké-Agbo, Jacques Zola-Sahossi, Horace Dègnonvi, Albert Salako, Michel Sèzonlin, Martin C Akogbéto

**Affiliations:** 1 Laboratoire Evolution, Biodiversité des Arthropodes et Assainissement, FAST, University of Abomey-Calavi, Cotonou, Bénin; 2 Centre de Recherche Entomologique de Cotonou (CREC), Cotonou 06BP2604, Benin

**Keywords:** *Anopheles gambiae s.s*, Pyrethroid resistance, Long-lasting insecticidal net, Washing, Effectiveness, Experimental hut, Benin

## Abstract

**Background:**

An investigation carried out in Benin has shown that, in some areas close to rivers where density of mosquitoes is high, long-lasting, insecticidal bed nets (LLINs) are permanently used. In such areas, LLINs are washed every month. Based on this situation, the 20-wash minimum efficacy advised by the manufacturers would be inadequate. The main goal of this study was to evaluate the effectiveness of LifeNet®, Olyset® and Permanet® 2.0 washed several times against *Anopheles gambiae sensu stricto (s.s.)* populations, which have developed high resistance to pyrethroids.

**Methods:**

Efficacy of LifeNet®, Olyset® and PermaNet® 2.0 washed 30 and 40 times was expressed in terms of blood-feeding inhibition rate, deterrence, induced exophily and mortality rates. This WHOPES phase II evaluation, conducted in experimental huts in Akron (southern Benin) and in Malanville (northern Benin), was accompanied by WHOPES Phase I evaluation.

**Results:**

Over 40 successive washes, LifeNet® induced a mortality rate over 80% in phase I. However, beyond 10 washes, Permanet® 2.0 and Olyset induced dramatically reduced mortality rates, respectively 12.5 and 2.5%. With regard to Phase II results, unwashed LifeNet®, LifeNet® and Olyset® washed 30 and 40 times induced a similar exophily rate per study site (at least 58% in Malanville and at least 71% in Akron). Regarding blood feeding inhibition, LifeNet® and Olyset® washed 30 and 40 times significantly reduced wild *An. gambiae s.s.* blood feeding showing a similar personal protection as unwashed LifeNet®. LifeNet® washed 30 and 40 times induced mortality rates significantly higher than those induced by Olyset® and Permanet® 2.0 (P < 0,05).

**Conclusion:**

LifeNet®, followed by Olyset®, have shown good efficacy against host-seeking resistant *An. gambiae s.s.* population in experimental huts in Benin. Lifenet® have shown to be an effective and promising vector control tool to prevent malaria in areas where repeated washings is a common practice in the community.

## Background

Malaria remains an obstacle for the development of sub-Saharan African countries, therefore, the good will of ministries of health and effective strategies are required to roll it back. Vector control programmes in African countries are based on mosquito control targeting strategies, especially the use of long-lasting insecticidal nets (LLINs) and indoor residual spraying (IRS). Both methods have proved very effective for malaria control. Clarke *et al.*[1] noted in 48 villages a reduction of 51% in the prevalence of *Plasmodium falciparum* in children sleeping under mosquito nets in good condition, compared to children without mosquito nets. In Bioko Island in Equatorial Guinea, the simultaneous use of IRS, LLINs and artemisinin combination therapy (ACT) resulted in a 90% drop in the presence of circumsporozoite antigen of *P. falciparum* in *Anopheles gambiae,* from 2003 to 2007. During the same period, malaria parasitaemia in children aged under five years fell from 42 to 18% and a 70% mortality drop [2].

Insecticide-treated (mosquito) nets (ITNs) have been used for malaria control in sub-Saharan Africa for over 20 years. However, it has been only six years since the latest models, the LLINs, have been widely distributed through large scale, free, public distribution campaigns [3].

LLINs are the main and best vector control tool to prevent malaria today. WHO guidelines state that LLINs should have adequate insecticidal activity after 20 standard washes and a serviceable life duration of minimum of three years [4]. Based on these criteria, the WHO Pesticide Evaluation Scheme (WHOPES) has given approval to 13 LLIN products [5,6]. One of the challenges faced with LLINs today is the appearance of pyrethroid resistance in *Anopheles* and the reduction of serviceable life*,* particularly in West Africa [7,8]. In addition to the emergence of pyrethroid resistance, high frequency washing of LLINs has been observed in some areas of high density mosquitoes and high use of LLINs. An investigation conducted in Benin showed that in areas located nearby rivers, LLINs are washed every month (equivalent to 36 washings over three years) (Akogbéto *et al*., unpublished data). The present study aimed to evaluate the effectiveness of three LLINs (LifeNet®, OlysetNet® and Permanet® 2.0) after multiple washes against *An. gambiae sensu stricto (s.s.)* populations, which have developed high resistance to pyrethroids. These LLINs recommended by WHO were tested against resistant *An. gambiae s.s.* in Benin in experimental huts. The efficacy of each LLIN was measured using blood-feeding inhibition, deterrence, induced exophily and mortality. The trials were conducted in verandah-trap huts in Akron (southern Benin) and Malanville (northern Benin) where *An. gambiae s.s.* has developed high resistance to insecticides. The main resistance mechanism reported in the two areas is *Kdr* mutation with more than 80% frequency in Akron and over 70% in Malanville. The type of experimental huts used in this study simulates domestic habitations. *An. gambiae* s.s. M form was the target species. Results of this study will serve as a baseline to the national malaria control programmes for the proper choice of LLIN candidates to control malaria vectors where mosquitoes have developed resistance to pyrethroids and repeated washings practices have been noted in the community.

## Methods

### Long-lasting insecticidal nets

PermaNet® 2.0 (Vestergaard Frandsen SA) is a LLIN made of multifilament polyester (75–100 denier) fabric, factory coated with a wash-resistant formulation of deltamethrin at a target dose of 1.8 g/kg (55 mg/m^2^). Olyset Net® (Sumitomo Chemicals, Osaka, Japan) is a LLIN made of knitted polyethylene (>150 denier) thread with permethrin at 20 g/kg (2% w/w), incorporated into the polyethylene fibres during the manufacturing process. The durability as advised by the manufacturer is five years (minimum) from the first use. Lifenet (Bayer CropScience) is a LLIN made of 100% polypropylene treated with deltamethrin (0.85% w/w), incorporated into fibres during the manufacturing process. The useful life of Lifenet is > five years when used as directed.

### Design of huts used

The trials were conducted in Akron (southern Benin) and Malanville (northern Benin) and the huts, built according to WHO guidelines [6], were made from concrete bricks, with a corrugated iron roof, a ceiling of thick polyethylene sheeting and a concrete base surrounded by a water-filled channel to prevent entry of ants. Mosquito access was via four window slits constructed from pieces of metal, fixed at an angle to create a funnel with a 1-cm wide gap. Mosquitoes fly upward to enter through the gap and downwards to exit, which precludes or greatly limits exodus though the aperture enabling the majority of entering mosquitoes to be accounted for. A single verandah trap made of polyethylene sheeting and screening mesh, measuring 3 m long, 2.5 m wide and 1.5 m high, projected from the back wall of each hut. Movement of mosquitoes between hut and verandah was unimpeded during the night.

### WHO susceptibility bioassay prior the implementation of the evaluation

At the beginning of the study, larvae and pupae of *Anopheles* species were collected from breeding sites in Akron and Malanville and kept in separate, labelled bottles related to each locality. The samples were reared to adult stage at the insectary of CREC (Centre de Recherche Entomologigue de Cotonou) for WHO tube bioassay. Adult female mosquitoes aged two to three days were exposed to the diagnostic dose of permethrin, deltamethrin and bendiocarb (this other insecticide class was used as positive control) for susceptibility tests for one hour, using insecticide-impregnated papers as described by the standard WHO testing protocol [9]. The insecticide-free papers were used as control. After exposure, mosquitoes were transferred to control tubes and maintained on 5% honey solution. The survivors after 24 hours’ DDT exposure were retained on silica gel for standard PCR.

### Study design

Adult volunteers were recruited to sleep in the huts under the nets, and mosquitoes were collected the next morning. Participants were recruited from the inhabitants of the two locations (Akron and Malanville). Pregnant and breast-feeding women were not included in the study. After having announced through the district that this project was looking for volunteers to sleep under the nets, a selection was carried out with the approval of the traditional head of the district. The volunteers were informed of the objectives of the study and signed (through a literate witness if illiterate) an informed consent.

Sleepers were rotated randomly among huts each night of the study. They entered the huts at dusk at 21:00 hours and remained inside until dawn (06:30 hours). In the morning, dead mosquitoes were collected from the floor of the huts using forceps; while resting mosquitoes were collected from the walls, the roofs of the huts and exit traps using mouth aspirators. Mosquitoes were scored by as dead or alive and as fed or unfed by location collected inside the huts. All mosquitoes collected alive were placed in plastic netted cups and fed with 5% honey solution for 24 hours holding to assess delayed mortality.

Six experimental huts were used in Malanville as follow:

One hut for LifeNet® washed 30 times

One hut for LifeNet® washed 40 times

One hut for Olyset® washed 30 times

One hut for Olyset® washed 40 times

One hut for unwashed LifeNet® (positive control)

One hut for untreated net (negative control)

Eight experimental huts were used in Akron as follow:

One hut for LifeNet® washed 30 times

One hut for LifeNet® washed 40 times

One hut for Olyset® washed 30 times

One hut for Olyset® washed 40 times

One hut for Permanet® 2.0 washed 30 times

One hut for Permanet® 2.0 washed 40 times

One hut for unwashed LifeNet® (positive control)

One hut for untreated net (negative control)

LLINs were rotated randomly among huts each week according to Latin square of the study. Entomological parameters measured in experimental huts according to the duration of the trial were:

Deterrence due to each LLIN (reduction of mosquitoes in hut entry relative to the control hut fitted with untreated net);

Induced exophily (the proportion of mosquitoes that exited early and were found in exit traps) due to each LLIN;

Blood-feeding inhibition (the number of blood-fed mosquitoes relative to the control hut). It also expressed the personal protection, which was calculated as follows: 100(B_u_-B_t_)/B_u_ where B_u_ is the number of blood-fed mosquitoes in the untreated net hut and B_t_ is the number of blood-fed mosquitoes in the treated nets huts;

Immediate and delayed mortality of mosquitoes (the proportion of mosquitoes that were killed).

### Washing process

The nets were washed at CREC according to a protocol adapted from the standard WHO washing procedure used in phase I trials [4]. Nets were washed in non-plastic bowls (aluminium) containing 10 l of well water with a maximum hardness of 5 dH and containing 2 g/l soap (“savon de Marseille”) using manual agitation. Each net was agitated for 6 min within a total of 10 min washing/soaking period. Agitation was done by stirring the net with a pole at 20 rotations per min. Rinsing was done twice using clean water (10 l per rinsing, i e, 20 l/net). Nets were dried horizontally in the shade then stored at a mean of 30°C temperature.

### Chemical analyses: cone bioassay and tunnel tests

#### *Cone bioassay test*

Bioassays were done according to the WHO procedures for cone tests [4]. Cones were placed on the net. Eight to ten females of *An. gambiae s.s.* susceptible reference strain Kisumu were introduced per cone and exposed for 3 min to treated nets and to the control net. After exposure, the mosquitoes were held for 24 hours with access to honey solution. Bioassays were replicated, and then at least 80 mosquitoes on each net were tested per LLIN. Mosquitoes exposed to untreated nets are used as controls. Bioassays were carried out at 25+/-2°C and 70+/-10%. Knock-down was measured after 60 min post-exposure and mortality after 24 hours.

#### *Tunnel test*

*Anopheles gambiae s.s.* larvae were collected at Akron and reared to five to eight-day old adult mosquitoes. The essay was carried out in laboratory by releasing non-blood-fed female anopheline (at least 30 to 40 specimens) mosquitoes at 19:00 in one of the compartments (compartment A) of a 60-cm tunnel (25 cm × 25 cm square section) made of glass. At one third of the length of the glass tunnel, a disposable cardboard frame was placed with a holed piece (nine holes each of 1 cm in diameter) of each LLIN. A guinea pig was placed in the second compartment (compartment B). The next day morning at 07.00, the dead mosquitoes, the survivors, the fed and unfed mosquitoes in the compartments A and B were collected and analysed. Data obtained in the tunnel containing the holed piece of the impregnated nets (25 × 25 cm) were compared to the control.

The impact of each LLIN was measured in terms of:

Deterrence or penetrating rate (reduction in compartment B entry relative to the control compartment B fitted with untreated net);

Blood-feeding inhibition (the reduction in blood feeding compared with that in the control tunnel);

Immediate mortality: proportion of mosquitoes collected dead after contact with the treated material.

Delayed mortality: proportion of mosquitoes collected alive after contacted with the treated material and recorded dead after 24 hours holding.

Overall mortality: the sum of immediate and delayed mortality.

### Perceived side effects

The sleepers under the nets were questioned at the end of the experiment about perceived adverse or beneficial side effects of the treated nets.

### Statistical analysis

After the intervention began, the number of mosquitoes of each species entering the huts, the proportion of mosquitoes that exited early, the proportion that were killed within the hut and the proportion that successfully blood fed were compared by species and were analysed using Poisson regression for numeric data and logistic regression for proportional data (e g, STATA 6 Software). The clustering of observations made in one hut-night and any variation between huts and sleepers were controlled and comparisons between LLINs were made by successively dropping LLINs from the overall comparison, and this process allowed each LLIN to be compared with every other one.

### Ethical clearance

Approval (N° 007) was obtained from the ethics committee of the Benin Ministry of Health. Written informed consent was obtained from the volunteers who slept in the experimental huts to attract mosquitoes. In case of any symptoms, they were admitted at the nearest Primary Health Centre.

## Results

### Status of vector resistance at the beginning of the study in the two experimental hut stations: Akron and Malanville

According to [9] criteria, *An. gambiae s.s.* population from both study sites was resistant to permethrin and deltamethrin (mortality rate <80%). However, this same mosquito population was susceptible to bendiocarb. Concerning deltamethrin resistance in Malanville, the emergence is recent and its spread has been very fast: 23.71% mortality rate in 2012 (Table 1) compared to 99% in 2004. In 2004, Malanville was declared the only area in Benin where *An. gambiae s.s.* is susceptible to pyrethroids. After molecular characterization by PCR, the samples of *An. gambiae s.s.* population analysed were M molecular form. The main investigated resistance mechanism in the two localities is *kdr* mutation with a frequency of 74% in Akron and 90% in Malanville (Table 1).

**Table 1:** Status of vector resistance at the beginning of the study in two experimental hut stations in Akron and Malanville

**Susceptibility test**					***Kdr *** **mutation**			
**Tested insecticide**	Localities	Tested number	Mean mortality rate	95% CI	RR	RS	SS	F (Kdr)
**Bendiocarb 0.1%**	Akron	99	100	[96.33-100]	21	17	2	0.74
	Malanville	93	100	[96.11-100]	38	10	0	0.90
**Permethrin 0,75%**	Akron	64	39.06	[27.1-52.07]	21	17	2	0.74
	Malanville	97	30.93	[21.93-41.12]	38	10	0	0.90
**Deltamethrin 0.05%**	Akron	61	70.49	[57.43-81.48]	21	17	2	0.74
	Malanville	97	23.71	[15.66-33.43]	38	10	0	0.90

95% CI: Significant threshold confidence limit.

RR: homozygote kdr resistant alleles.

SS: homozygote kdr susceptible alleles.

RS: heterozygote kdr alleles.

F (kdr): Frequency of kdr mutation.

### Culicidae diversity in the two experimental hut stations

**Figure 1:**
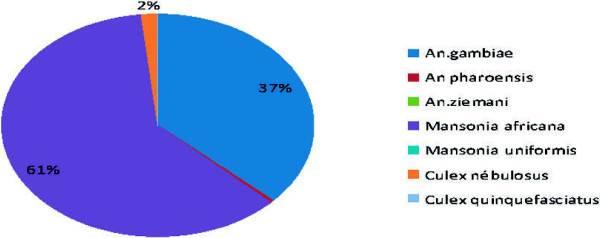
Mosquito species composition in Malanville.

**Figure 2:**
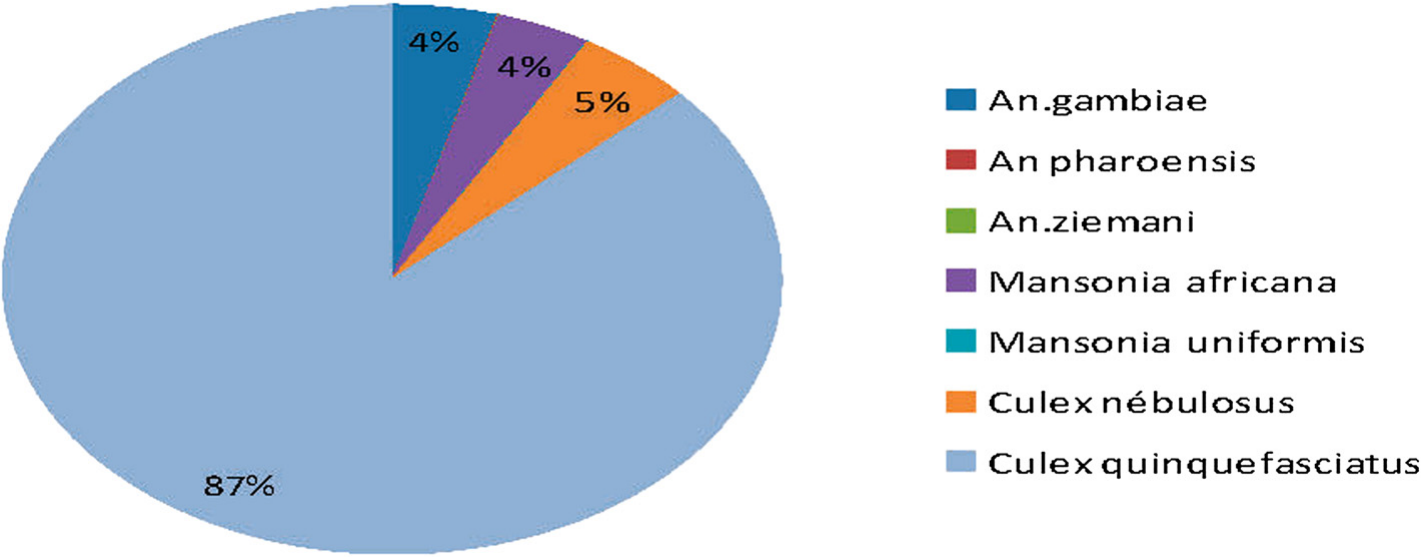
Mosquito species composition in Akron.

During the WHOPES phase II evaluation, the mosquito species present were recorded daily in each experimental hut station in Akron and Malanville. Various species of mosquitoes were collected. Figures 1 and 2 show the most common mosquitoes collected. In Malanville, two major species were collected: *Mansonia africana* (61.09%) and *An. gambiae s.s.* (36.84%); in Akron: *Culex quinquefasciatus* (86.75%), *Culex nebulosus* (4.84%), *An. gambiae s.s.* (4.38%), *Mansonia africana* (3.93%), and *Anopheles ziemanni* (0.01%).

### Laboratory trials on the effectiveness of the three nets washed one to 40 times

#### *Bioassay results*

**Figure 3:**
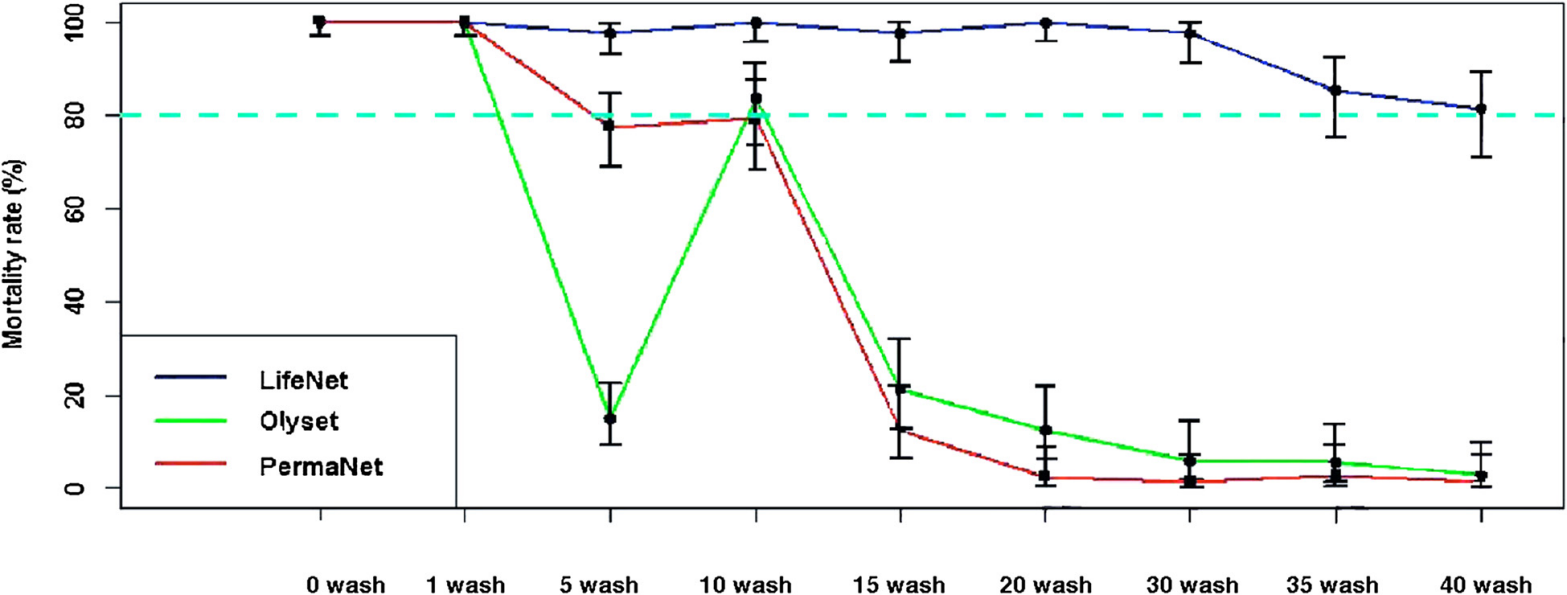
**Mortality (%) of laboratory susceptible strain “Kisumu’ exposed to pieces (25 cm x 25 cm) of Olyset net, LifeNet and Permanet 2.0 after successive washes, using cone bioassay.** 95% Confidence Interval with 80% as threshold show statistical significance.

The bio-efficacy of the three LLINs (LifeNet®, Olyset® and Permanet® 2.0) was performed after nine series of washing (one, five, ten, 15, 20, 25, 30, 35 and 40 washes). The unwashed LifeNet was used as positive control. About 50 specimens of Kisumu *An. gambiae* susceptible strain were exposed to each washed and unwashed net. More than 2,000 specimens of susceptible strain *An. gambiae* were analysed. Figure 3 shows the effectiveness of each net. According to WHO [4], the threshold of bio-efficacy is 80% mortality or 95% knock-down of mosquitoes exposed. All nets therefore giving less than 80% mortality after 24 hours of the holding time, or less than 95% knock-down after 60 min of exposure are considered ineffective. Based on these criteria, LifeNet® was the only LLIN which was effective during all washings: more than 97% of susceptible strain *An. gambiae* were dead after exposure to LifeNets® washed one to 30 times, 85.33 and 81% to LifeNets® washed 35 and 40 times, respectively. Until 30 washings, LifeNets® were as effective as control unwashed LifeNet® (no wash). In Permanet® 2.0 and Olyset®, the effectiveness was observed at ten washes. Beyond ten washes, the mortality rates were too low: 12.5 and 2.5% for Olyset® and Permanet®, respectively, washed 20 times (Figure 3). In parallel, the knock-down effect was found until ten washes for LifeNet®, five washes for Permanets® and only one wash for Olyset® (Table 2).

**Table 2:** **Percentage of laboratory-susceptible strain *Anopheles gambiae *** **Kisumu knocked down after 60 minutes’ exposure to pieces (25 x 25cm) of Olyset, LifeNet and Permanet 2.0 nets washed one to 40 times using cone bioassay**

	**0 wash**	**1 wash**	**5 washes**	**10 washes**	**15 washes**	**20 washes**	**30 washes**	**35 washes**	**40 washes**
**LifeNet**	**100**	**99.17**	**100**	**96.25**	78.75	95	87.34	76	83.75
**Olyset**	**100**	**100**	70	88.61	27.5	31.25	4.41	5.56	4.29
**Permanet 2.0**	**100**	**100**	95	49.35	17.12	8.75	6.85	5.33	5.48

95% CI: Significant threshold confidence limit.

### Tunnel test results

The tunnel test results are summarized in Table 3. Larvae of *An. gambiae s.s.* collected around Akron station and reared to two to five-day old adults were used to perform tunnel tests. The study did not find it necessary to use *An. gambiae s.s.* from Malanville because the Akron and Malanville populations are considered to be the same: both are *An. gambiae s.s.* and characterized by more than 70% *kdr* mutation. The three types of LLINs (LifeNet®, Olyset® and Permanet® 2.0) washed one, ten, 20, 30 and 40 times were analysed. Untreated net were the control. The effectiveness of each LLIN was evaluated in terms of: penetrating rate (percentage of mosquitoes going through the holes in the piece of each LLIN and penetrating compartment B with a guinea pig), *An. gambiae s.s.* feeding rate on the guinea pig and mortality rate (immediate mortality and overall mortality after 24 hours holding time).

**Table 3:** **The efficacy of LifeNet, Olyset and Permanet 2.0 against *Anopheles gambiae *** **collected from Akron determined by tunnel test**

		**Nb tested**	**Penetrating rate**	**95% CI**	**Blood-feeding rate**	**95% CI**	**% immediate mortality**	**% overall mortality**	**95% CI**
	Untreated LifeNet	49	77.55	[65.87-89.23]	79.59	[68.31-90.88]	0	0	[0–7.27]
**1 wash**	LifeNet	45	8.89	[0.57-17.20]	2.22	[0.39-11.57]	100	100	[92.13-100]
	Olyset	45	4.44	[1.58-10.47]	0	[0–7.87]	64.55	79.74	[68–91.49]
	Permanet 2.0	47	14.89	[4.72-25.07]	0	[0–7.56]	80.60	92.73	[85.30-100.15]
**10 washes**	LifeNet	47	0	[0.00-0.00]	0	[0–7.56]	68.48	78.18	[66.37-89.99]
	Olyset	49	10.2	[1.73-18.68]	4.08	[1.13-13.71]	48.84	58.14	[44.33-71.95]
	Permanet 2.0	48	16.67	[6.12-27.21]	4.17	[1.15-13.98]	66.76	78.63	[67.04-90.23]
**20 washes**	LifeNet	48	50	[35.85-64.15]	6.25	[2.06-16.22]	97.63	97.63	[93.32-101.93]
	Olyset	27	29.63	[12.41-46.85]	0	[0–12.46]	87.34	91.56	[81.07-102.05]
	Permanet 2.0	42	35.71	[21.22-50.21]	28.57	[14.91-42.23]	53.88	59.30	[44.44-74.16]
**30 washes**	LifeNet	51	23.53	[11.89-35.17]	1.96	[0.84 - 7.77]	91.06	95.53	[89.86-101.20]
	Olyset	43	11.63	[2.05-21.21]	2.33	[0.41-12.06]	33.75	44.35	[29.50-59.20]
	Permanet 2.0	49	57.14	[43.29-71.00]	36.73	[23.24-50.23]	32.56	41.86	[28.05-55.67]
**40 washes**	LifeNet	57	28.07	[16.40-39.74]	12.28	[3.76-20.80]	88	94	[87.84-100.17]
	Olyset	50	12	[2.99-21.01]	0	[0–7.13]	70.37	84.05	[73.90-94.20]
	Permanet 2.0	55	63.64	[50.92-76.35]	89.09	[80.85-97.33]	0	0.55	[0.32-9.61]

Parameters were calculated after 24 hours’ observation.

95% CI calculated indicates the level of difference between each net.

Adult female aged from five to eight days were used.

### Penetrating rate

Penetration through the holes in the untreated net was easy for mosquitoes. Untreated net (negative control) was therefore not a difficult barrier to cross: 77.5% of *An. gambiae s.s.* had penetrated compartment B (Table 3). For the treated LLINs, the penetration rate was significantly lower compared to the control. Less than 10% of the mosquitoes succeeded in penetrating the holed LifeNet® and Olyset® washed one to ten times. The rate varied from 11.53 to 50% for the three LLINs washed 20–30 times. After 40 washes, the penetrating rate was low for the LifeNet® (28.07%) and Olyset® (12%). For Permanet® washed 40 times (63.64%), there was no difference compared to the control (untreated net), indicating the lowest level of protection.

### Blood-feeding inhibition

The blood-feeding rate of *An. gambiae s.s.* was high in the control tunnel provided with the holed, untreated net (79.59%). After one to 20 washes, the blood-feeding rate decreased to 0–6.25% for the three LLINs, except Permanet® 2.0 which had a blood-feeding rate of 28.57% after 20 washes. After 40 washes, the blood-feeding rates observed were low, 12.28 and 0% respectively for LifeNet® and Olyset®, but for Permanet® 2.0 the blood-feeding rate was much higher (89.09%), higher even than the control (79.59%) (Table 3).

### Mortality rate

During the study, less than 5% *An. gambiae s.s.* exposed to the untreated net were dead. After 40 washes, more than 80% of mosquitoes exposed to LifeNet® and Olyset® were killed except after ten washes (78.18% for LifeNet® and 58.14% for Olyset®) and after 30 washes for Olyset® (44.35%) (Table 3).

In conclusion, the knock-down effect of deltamethrin on the surface of LifeNet® limited the access of more than 70% of *An. gambiae s.s.* to the guinea pig. On the other hand, the knock-down effect of permethrin was high in the tunnels provided with holed Olyset®. In addition, inhibition of blood feeding and the lethal action of LifeNet® and Olyset® were still effective at 40 washes.

### WHOPES Phase II trials: effectiveness of the three LLINs washed one to 40 times in experimental huts

#### *Variation in density of host seeking mosquitoes’ per hut before implementation of LLINs*

The homogeneity attractiveness of the experimental huts was verified taking into account the numbers of mosquitoes caught in each hut prior to starting the evaluation (before treating the huts with the various insecticides, mosquito collections were done inside to measure the individual attractiveness of each hut for mosquitoes). This comparison showed that the individual huts did not differ significantly in terms of the number of mosquitoes entering the huts in Akron and Malanville, however, there is some difference, probably due to the position of each hut in relation to the mosquito larvae breeding sites. This difference was eliminated as sleepers and LLINs were rotated randomly according to each Latin square rotation among huts during each week of the study.

#### *Induced exophily*

The natural exophily of freely host-seeking *An. gambiae s.s.* in contact with untreated net was 47.92% in Akron. The rate was significantly different in huts provided with the positive control (unwashed LifeNet): 71.43% (p = 0.002). Similar results were obtained in Malanville: 31.39% for the natural exophily and 58.88% in contact with unwashed LifeNet®: p < 0.001 (Table 4). In huts provided with LifeNets® washed 30 and 40 times in Akron, the exophily rates were respectively 81.16 and 72.73%. A similar observation was made in Malanville: respectively 58.88 and 54.88%. These rates are significantly higher than was registered in the negative control hut (Table 4). LifeNet® washed 30 and 40 times showed similar exophily rates compared to unwashed LifeNet® both in Malanville and in Akron (P > 0.05). The same result was observed with Olyset® washed 30 and 40 times in Malanville. Olyset® and Permanet® 2.0 washed 30 and 40 times induced the same level of exophily as untreated net (negative control) in Akron (Table 4).

**Table 4:** Summary of experimental hut trial results for Anopheles gambiae at Akron and Malanville field stations

**Net treatment**	**Total tested/collected**	**% deterency (95% CI)**	**% exophily (95% CI)**	**% blood-fed (95% CI)**	**Personal protection (no. Fed)**	**% immediate mortality (95% CI)**	**% overall mortality (95% CI)**
**AKRON**							
**Untreated net**	144	-	47.92 -	26.39 -	-	14.58 [0.16-0.64]	15.97 [0.12-0.47]
**Unwashed LifeNet**	63	56.25	71.43 [1.44-5.21]	15.87 [0.23-1.12]	73.63	34.92 -	44.44 -
**LifeNet 30 X**	69	52.08 [0.53-1.35]	81.16 [2.38-9.53]	17.39 [0.28-1.19]	68.42	34.78 [0.48-2.05]	47.83 [0.57-2.29]
**LifeNet 40 X**	55	61.81 [0.78-2.02]	72.73 [1.48-5.81]	09.09 [0.09-0.72]	86.84	41.82 [0.63-2.84]	47.27 [0.54-2.33]
**Olyset 30 X**	37	74.31 [1.37-3.71]	59.46 [0.76-3.37]	29.73 [0.51-2.59]	71.05	21.62 [0.19-1.30]	21.62 [0.13-0.86]
**Olyset 40 X**	40	72.22 [1.24-3.31]	65.00 [0.98-4.25]	20.00 [0.28-1.62]	78.95	32.50 [0.38-2.09]	35.00 [0.29-1.53]
**Permanet 30 X**	79	45.14 [0.40-1.02]	56.96 [0.83-2.51]	32.91 [0.75-2.49]	31.58	32.91 [0.45-1.85]	36.71 [0.37-1.43]
**Permanet 40 X**	103	28.47 [0.19-0.51]	45.63 [0.55-1.31]	38.83 [1.03-3.05]	-5.26	16.50 [0.18-0.77]	18.45 [0.14-0.58]
**MALANVILLE**							
**Untreated net**	911	-	31.39 -	15.48 -	-	00.99 [0.02-0.06]	01.65 [0.01-0.03]
**Unwashed LifeNet**	676	25.8	58.88 [2.54-3.85]	05.47 [0.21-0.46]	73.76	23.22 -	44.97 -
**LifeNet 30 X**	563	38.2 [1.46-2.17]	54.88 [2.14-3.30]	07.99 [0.33-0.67]	60.09	17.23 [0.52-0.91]	30.37 [0.42-0.68]
**LifeNet 40 X**	679	25.47 [0.80-1.21]	59.20 [2.58-3.90]	07.95 [0.34-0.65]	61.70	19.15 [0.60-1.02]	36.23 [0.56-0.86]
**Olyset 30 X**	581	36.22 [1.34-1.99]	48.71 [1.67-2.57]	10.84 [0.48-0.91]	55.32	13.25 [0.37-0.68]	29.43 [0.40-0.64]
**Olyset 40 X**	484	46.87 [2.08-3.09]	53.72 [2.02-3.18]	11.57 [0.51-0.99]	60.28	17.77 [0.53-0.95]	26.86 [0.35-0.58]

-: means reference rate.

#### *Deterrency rate*

In both study sites, all treatment arms significantly induced the reduction of *An. gambiae s.s.* entry when comparing the reduction rates to those of untreated nets. Olyset® washed 30 and 40 times and LifeNet® washed 30 times induced a high significant reduction of entry compared to unwashed LifeNet (positive control) (Table 4).

#### *Blood-feeding rate*

The blood-feeding rate was 15.48% in huts fitted with the control net (untreated net) in Malanville and 26.39% in Akron. These two rates were higher compared to the huts fitted with the positive control (unwashed LifeNet®) (Table 4). In Malanville, only LifeNet® unwashed, washed 30 and 40 times induced a higher blood-feeding inhibition: with blood feeding rates of only 5.47, 7.99 and 7.95%, respectively. Olyset® washed 30 and 40 times (respectively, 10.84 and 11.57 of blood feeding) also significantly reduced *An. gambiae s.s.* blood-feeding rate compared to control. In Akron, Olyset® washed 30 times and Permanet® 2.0 washed 30 and 40 times induced a higher blood-feeding rate compared to untreated net, though this difference was not significant (Table 4). LifeNet® washed 40 times reduced blood-feeding rate significantly compared to the untreated net and all Olyset® and Permanet® samples. This result was not significantly different from unwashed LifeNet® and LifeNet® washed 30 times.

#### *Mortality rate*

In Malanville, immediate mortality rates induced by LifeNet® washed 30 times and Olyset® washed 30 and 40 times were significantly lower than those of unwashed LifeNet® (P < 0.05) (Table 4). In Akron, a similar result was observed with Permanet® 2.0 washed 40 times. However, the induced mortality by LifeNet® and Olyset® washed 30 and 40 times was not significantly different compared to unwashed LifeNet® (p > 5%).

Overall mortality rates induced by LifeNet® and Olyset® washed 30 and 40 times were significantly lower compared to unwashed LifeNet® (P < 5%) in Malanville (Table 4). In Akron, Olyset® and Permanet® washed respectively 30 and 40 times also induced an overall mortality significantly lower compared to unwashed LifeNet® (P < 5%) (Table 4).

#### *Side effects of the treatment on sleepers*

A regular follow up of side effects of the insecticide treatments on sleepers was conducted using a questionnaire. These include one or more of the following: itching of skin, facial burning/tingling, paraesthesia (numbness or a loss of physical sensation and/or tingling of skin), sneezing, liquid discharge from nose, feeling of headache, nausea, eye irritation and tears, experience of bad smell, body rashes etc. The only effect mentioned by sleepers during the three first day of in hut arm for unwashed LifeNet (positive control) was itching of skin. However, the sleepers noticed that the treatments reduced the biting nuisance of mosquitoes in the treated huts than in their own homes or the negative control hut. In response to the question “Would you like to continue the experiment?” they all responded “yes.”

## Discussion

The current study evaluated the impact of repeated washing on the effectiveness of three types of LLINs (LifeNet®, OlysetNet®, PermaNet® 2.0) under semi-field conditions in two West African experimental huts stations where *An. gambiae s.s.* is resistant to pyrethroids. After evaluation, Lifenet® followed by Olyset® showed the best efficacy, but Lifenet® was a good vector tool in areas where the frequency of washing was high.

Despite repeated washing, the exophily of *An. gambiae s.s.* recorded with the three LLINs was higher than the control net. Similar results were observed with Olyset® in Ivory Coast by N’guessan *et al*. [3], Hodjati and Curtis [10] and Chandre *et al.*[11]. It is obvious that repeated washing of LLINs can result in a reduction in efficacy due to loss of insecticide [12,13]. The excito-repellent effect of the deltamethrin (LifeNet®, Permanet®) and of permethrin (Olyset®), which explains the increase of the exophily, appeared differently in the three LLINs., by considering data from the experimental huts and the tunnel tests, it can be concluded that repeated washings does not completely affect the repellent effect. Indeed, during the tunnel tests, a high proportion of *An. gambiae s.s.* did not succeed in penetrating the pieces of the three types of LLIN. Given that *Anopheles* released in the tunnel belong to a population of mosquito resistant to pyrethroids, it is possible that those which succeeded are those carrying resistant genes. In a recent study from “Centre de Recherche Entomologique de Cotonou”, Gnanguenon *et al*. [14], showed that *An. gambiae* specimens carrying *kdr* resistant genes, especially the RR genotypes (resistant homozygotes), were more likely to pass through the holes of LLINs than the susceptible homozygotes SS. As all LLINs were washed in the same conditions according to WHO protocol guideline [4], the washing alone cannot therefore induce the reduction of excito-repellency observed to allow some *An. gambiae s.s.* to penetrate through the holes of LLINs. The level of resistance developed by *An. gambiae s.s.* to pyrethroids, which is very high in study sites is another factor.

Regarding the blood-feeding inhibition induced by LLINs, a relatively good performance in preventing mosquitoes from feeding was observed with LifeNet® and Olyset® after repeated washing, except Permanet ®2.0. Despite mosquito collectors being under LLINs, some mosquitoes succeeded in taking blood meal. Many phase II and phase III evaluations implemented in Benin, have shown that, in spite of houses being treated with insecticides, the majority of mosquitoes that enter these treated houses, succeed in taking blood meal before resting on treated walls [15,16]. Regarding bioassay results, before washing, all treated nets showed 100% knockdown (KD) and mortality, indicating full bioavailability of deltamethrin in the polypropylene (LifeNet®) and polyester (Permanet® 2.0) fibres, and of permethrin in the polyethylene (Olyset®) fibres. Despite multiple washing, LifeNet® was the only LLIN that was effective up 20 standard washes during all washing regimes. However, the efficacy of washed LLINs decreased with repeated washing. The short time (one day) interval between washes might not have given enough time for polyethylene- and polyester-based LLINs to regenerate, hence might resulted in the observed loss efficacy noted on Olyset® and Permanet® 2.0. Furthermore, the study conducted by Duchon *et al*. [17], concluded that the wash resistance of LifeNet® exceeded the minimum WHO requirements of 20 standard washes until 35 washes. Elsewhere, in a laboratory-based study carried out at CDC comparing wash resistance of six types of LLINs, mortality of less than 10% was recorded on Olyset® after only six washes using susceptible laboratory-reared *An.gambiae s.s.* in cone bioassay tests [18]. Considerable loss of Permanet® 2.0 efficacy was found in this study, using the interim WHO standard water bath protocol in phase I tests. Graham *et al*. [19] showed a far superior retention of efficacy after washing Permanet® 2.0 compared to the conventionally treated deltamethrin nets (CTDN), which were white polyester multifilament nets (SiamDutch Mosquito netting Co., Bangkok, Thailand). Among the three LLINs evaluated, Olyset® and Permanet® 2.0 have been on the market the longest and are the most studied. Most of the studies carried out comparing the bio-efficacy of the two market-dominant LLINs [12] have consistently shown that Olyset® is more wash durable and less bio-effective compared to Permanet®. This may be because of the treatment technology used and netting material (Duchon S: Regeneration, wash resistance and efficacy of long-lasting insecticidal mosquito nets (LifeNet) from Bayer CropScience against susceptible mosquitoes of *Anopheles gambiae*, umpublished report).

The results of this study showed that LifeNet® followed by Olyset® are more wash durable and bio-effective up 20 standard washes until 40 washes. This is important because an investigation in Benin showed that in some areas near rivers, LLINs are washed every month, (equivalent to 36 washings over three years) (Akogbeto *et al.*, unpublished data). Further studies are needed, especially with standard universal WHOPES conditions, to confirm the superior wash durability and bio-efficacy of LifeNet®, which obtained WHOPES interim recommendation for use in prevention and control of malaria.

## Conclusion

Despite multiple washings, LifeNet® and Olyset® have shown good efficacy against *An. gambiae s.s.* in Akron and Malanville in Benin. Lifenet® is a good vector control tool to prevent malaria in areas of repeated washings.

## Competing interests

The authors declare that they have no competing interests.

## Authors’ contribution

FRA, GGP and MCA designed the study. FRA, GGP, VG, JZ, HD, and AS carried out the experiments. FRA and FO analysed the data. FRA drafted the manuscript. FRA, MS and MCA critically revised the manuscript. All authors read and approved the manuscript.
